# Interactions between ALKBH5 and reader proteins in tumors: functions and molecular mechanisms

**DOI:** 10.3389/fonc.2025.1611007

**Published:** 2025-09-01

**Authors:** Jiahui Ou, Bingchen Liu, Yi Yu, Yingchun He, Yuyu Gao, Lingli Chen, Xia Chen, Huai Tao

**Affiliations:** ^1^ School of Medicine, Hunan University of Chinese Medicine, Changsha, Hunan, China; ^2^ Hunan Provincial Key Laboratory of Integrated Traditional Chinese and Western Medicine, Hunan University of Chinese Medicine, Changsha, Hunan, China; ^3^ Department of Orthopedics, The Second Xiangya Hospital of Central South University, Changsha, Hunan, China

**Keywords:** M6A, ALKBH5, reader proteins, tumor, m6A inhibitors

## Abstract

RNA methylation modifications are widespread in eukaryotes and prokaryotes, with N6-methyladenosine (m6A) methylation being the most prevalent internal modification in eukaryotic mRNA and having become a prominent focus of tumor research in recent years. Up to now, substantial evidence has suggested that the dysregulated RNA demethylase ALKBH5 can interact with m6A reader proteins to modulate a wide range of mRNA biological progress, including mRNA shearing, export, metabolism, and stability, ultimately influencing tumorigenesis and development. To deeply understand the regulatory roles of ALKBH5 and reader proteins in tumor progression, this review aims to summarize the structures of ALKBH5 and reader proteins, as well as their cooperative regulatory mechanisms that affect the occurrence and development of tumors originating from different systems. Furthermore, the potential applications of targeting ALKBH5 and reader proteins in antitumor drug development are summarized, hoping to provide a strong basis for advancing antineoplastic research in the future.

## 1 Introduction

**Figure 1:**
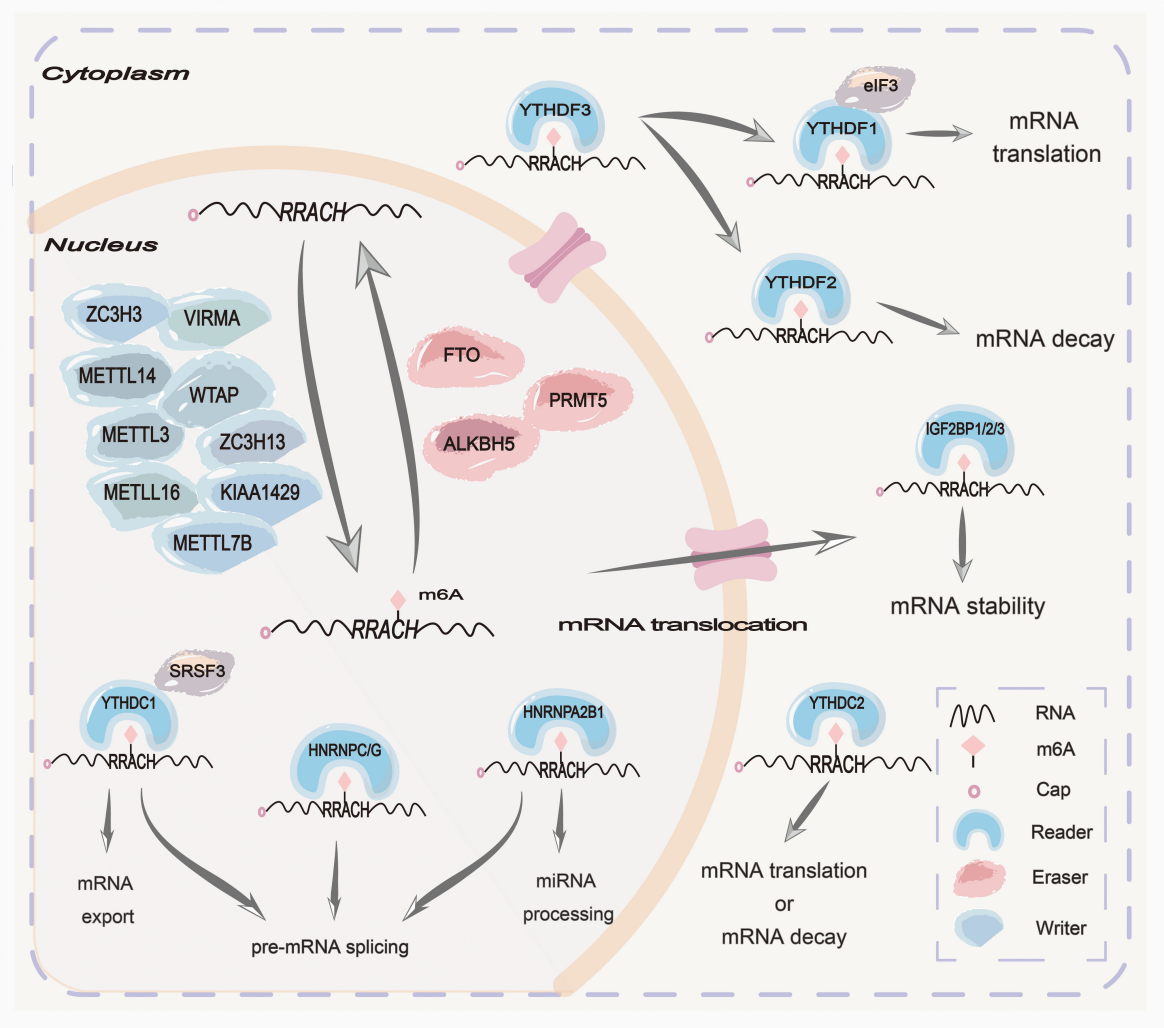
Mechanisms of m6A N^6^-methyladenosine(m6A) modification are summarized at the cellular and molecular levels. The m6A methylation complex consists of the core METTL3 and its adaptors to add m6A onto target mRNAs in the nucleus. Two main m6A demethylases, FTO and ALKBH5, can erase the methylation modification from target mRNAs in the nucleus. PRMT5 is a methyltransferase that inhibits m6A modification of mRNA by enhancing the nuclear translocation of ALKBH5. Some readers in the nucleus, such as HNRNPC/G, HNRNPA2B1, and YTHDC1, can mediate pre-mRNA splicing. Moreover, HNRNPA2B1 can mediate miRNA processing. Some readers in the cytoplasm, such as YTHDF1/2/3, IGF2BP1/2/3, and EIF3, can mediate mRNA translation, stabilization, and decay.

Epigenetics is modifying genetic changes in gene expression without altering the sequence of nuclear DNA, including DNA methylation, RNA methylation, histone modification, and protein methylation (1). N6-methyladenine(m6A), the methylation of the sixth nitrogen atom of adenine in RNA molecules by methyltransferase, is widely observed in various types of RNAs, including mRNAs, miRNAs, lncRNAs, circRNAs, tRNAs, and other protein-coding and non-coding RNAs, which is the most extensively studied post-transcriptional chemical modification to affect gene expression (2–6). This modification represents a dynamic biological process involving three distinct components: writers (methyltransferases), erasers (demethylases), and readers (m6A-binding proteins) (7) (**Figure 1**).

The m6A methyltransferase complex (MTC), often referred to as “writers”, is composed of a core heterodimer of METTL3-METTL14 and other significant ligands. These ligands, including WTAP, KIAA1429, ZC3H13, ZC3H3, VIRMA, METTL7B, and METTL16 are recruited to identify GAC and AAC sites in mRNA, thereby forming MTC to catalyze RNA methylation (8–11). However, the m6A demethylase, often referred to as “erasers”, comprises the alpha-ketoglutarate-dependent dioxygenase alkB homolog 1-8 (ALKBH1-8), fat mass and obesity-related proteins (FTO), and protein arginine methyltransferase 5 (PRMT5) (12, 13). Due to their distinct tissue distribution characteristics, m6A demethylases exhibit differences in substrates and functions. Among them, ALKBH5 has been reported to catalyze the removal of m6A in mRNA, ssRNA, and ssDNA, as well as the double methylated species m^6^_2_A in rRNA, which plays a significant role in embryo development, autophagy, and antiviral innate reactions (14). FTO has been shown to demethylate 3-methylamine (3-meT) in ssDNA and 3-methyl-uracil (3-meU) in ssRNA, m6A in mRNA, m6A_m_ in mRNA and snRNA, and m1A in tRNA, mainly affecting metabolic-related functions and diseases (14). PRMT5 is a new methyltransferase that inhibits m6A modification of RNA by enhancing the nuclear translocation of ALKBH5 (15). Additionally, the reader proteins can recognize RNA modifications to carry out a series of functions, including regulating the translation and stability of RNAs (16–19). The reader proteins are primarily comprised of the YT521B homology (YTH) domain family, insulin-like growth factor 2 mRNA binding proteins (IGF2BPs), and heterogeneous nuclear ribonucleoproteins (HNRNPs) (20, 21). In conclusion, methyltransferases, demethylases, and reader proteins play crucial roles in splicing, stabilization, translation, localization, and transportation, and even reversely regulate chromatin status. However, dysregulation of the above three components may result in tumorigenesis, metastasis, chemoresistance, and radio-resistance that potentially impact tumorigenesis and development, indicating their potential in therapeutic applications for tumors (22, 23).

With the deep research on the regulatory mechanisms of m6A modification, there has been a growing focus on the functional ALKBH5 and reader proteins in tumorigenesis and tumor development (24, 25). Both ALKBH5 and reader proteins can interact with each other to synergistically modify cellular processes by controlling the activities of RNA metabolism, which is related to the occurrence and development of various tumors (26). In the mechanism, the dysregulated expression level of ALKBH5 often regulates its targeted genes in an m6A-reader proteins-dependent manner (27). In this review, the mechanism of ALKBH5 interacting with reader proteins to regulate targeted gene expression and participate in tumor processes is summarized in different tumors based on the structure and function of ALKBH5 and reader proteins. Besides, the potential applications of targeting ALKBH5 and reader proteins in anticancer drug development are summarized to gain deeper insights for promoting research on antineoplastic agents in the future.

## 2 Structures and functions of ALKBH5 and reader proteins

### 2.1 ALKBH5

ALKBH5 is a member of the AlkB family, a 2-oxoglutarate (2OG) and ferrous acid-dependent nucleic acid oxygenase, which is located on human chromosome 17p11.2 (28). ALKBH5 possesses an active site motif HXDX*n*H (X = any amino acid) for binding Fe (II) and RX*n*R for binding 2OG and recognizing substrate (29). As a 2OG oxygenase, the catalytic core of ALKBH5 contains a double-stranded β-helix fold (DBSH) domain that consists of 11 *β* strands (*β*1–*β*11) and 5 *α* helices (*α*1–*α*5) (30, 31). The DSBH functions as a scaffold for three Fe (II)-ligating amino acids, including His204, Asp206, and His266, which constitute the well-conserved HXDX*_n_* H motif coordinating metal ions (30, 31). Through structural comparison, the primary distinguishing feature between ALKBH5 and other ALKB family proteins lies in the recognition cap located outside the DBSH fold, which is composed of two components referred to as “Flip1” and “Filp2” (30). Flip1 exposes an uncovered and relatively large space over the active site, and flip2 potentially determines the specificity of ALKBH5 in substrate selection, both of which play vital roles in substrate selectivity (30). Besides, the disulfide bond between Cys-230 and Cys-267 contributed to the specific selectivity of ALKBH5 towards single-stranded nucleic acid substrates (30).

The activity of ALKBH5 significantly influences mRNA exportation and RNA metabolism (25). Recently, an increasing amount of evidence has indicated that ALKBH5 can regulate the proliferation, metastasis, invasion, and apoptosis of tumor cells, as well as control the self-renewal ability of cancer stem cells (32–34), indicating that it has significant practical value in the diagnosis and prognostic assessment of cancer. For example, evaluating the expression level of ALKBH5 in blood or tissue samples from lung cancer patients has the potential to contribute to early diagnosis, pathological classification, and staging of lung cancer patients (35). Furthermore, researchers have utilized ALKBH5 to develop risk assessment models, which divide lung adenocarcinoma (LUAD) patients into high-risk and low-risk categories on the basis of the constructed models, emphasizing the potential of ALKBH5 as a marker for cancer prognosis (35, 36). Therefore, a deep understanding of the complex molecular structure and functions of ALKBH5 is essential for the strategic development of targeted activators or inhibitors for tumor therapy.

### 2.2 Structures and functions of reader proteins

#### 2.2.1 The YTH domain family protein

The YTH family proteins are the primary major “reader proteins” that directly bind to m6A sites on RNA. These proteins, consisting of approximately 100–150 amino acid residues, are present in various eukaryotes and are characterized by 14 conserved residues within an α-helix/β-sheet structure (37, 38). The first YTH protein, YT521-B, has a unique 100–150 residue domain known as the YTH domain, which is highly conserved among YT521-B homologs (39). In the YTH domain, a hydrophobic pocket formed by three highly conserved aromatic residues is called an aromatic cage, which has been detected to recognize RNA modified with N6-methyladenosine (39), suggesting that the YTH domain serves as a module for m6A recognition via a methylation-dependent manner.

The YTH domain-containing family proteins (YTHDFs) family includes three members called YTHDF1, YTHDF2, and YTHDF3 (39), which are composed of a C-terminal YTH domain and an N-terminal domain rich in P/Q/N (Pro/Gln/Asn). In the cytoplasm, YTHDF1 can recognize m6A-modified RNA and enhance its translation via its N-terminal domain (40). Interestingly, recent research has shown that YTHDF1 is capable of interacting with Argonaute 2 (AGO2) to stimulate the production of P-bodies for mRNA degradation (41). YTHDF2 is the most explored YTHDF and is generally expressed at higher levels than both YTHDF1 and YTHDF3 in most cells (42). The C-terminal domain and N-terminal domain of YTHDF2 respectively bind m6A-tagged mRNA and promote mRNA degradation in the cytoplasm (16). Additionally, YTHDF3 can promote m^6^A-mRNA translation by cooperating with YTHDF1 and interacting with the 40s/60s ribosome subunits (19). More interestingly, the YTHDF family forms a functional model: upon entering the cytoplasm, m6A-modified mRNAs are initially bound by the YTHDF3 or YTHDF3-YTHDF1 complex, and subsequently recognized by the YTHDF2, thus regulating the biological progress of targeted mRNA (43).

The YTH domain containing (YTHDCs) family includes YTHDC1 and YTHDC2. The YTH domain within YTHDC1 specifically recognizes the m6A modification and G(m6A)C sequence primarily in the nucleus, thereby accelerating the degradation of m6A-tagged transcripts to regulate selective splicing (44), subcellular localization, and nuclear export (45). Additionally, studies have demonstrated that YTHDC1 enables the formation of membraneless organisms like nuclear condensates to potentially affect gene expression and nucleocytoplasmic export via liquid-liquid phase separation (LLPS) (46). YTHDC2 plays a critical role in regulating m6A mRNA stabilization by utilizing its ATP-dependent RNA helicase activity to recognize m6A modification and facilitate the recruitment of RNA degradation (47).

Collectively, the specific roles of the YTH family proteins are principally determined by their levels of expression, spatial distribution, and posttranslational modifications, which are complex and require further investigation in the future.

#### 2.2.2 The IGF2BP family protein

Insulin-like growth factor-2 mRNA-binding proteins (IGF2BPs, also known as IMPs or VICKZs), including IGF2BP1, IGF2BP2, and IGF2BP3 (48), are composed of two RNA recognition motifs (RRM) domains (RRM-1 and RRM-2) of N-terminal and four heterogeneous nuclear ribonucleoprotein (hnRNP) K homology (KH) domains (KH-1 to KH-4) of C-terminal (49). The N-terminal RRM domains may mediate interactions with other RBPs (RNA-binding proteins) and affect the stability of RNA-protein complexes, and the C-terminal KH domains are responsible for recognizing and binding RNAs (50). IGF2BPs have the ability to combine with the m6A CU sequence of mRNA, thereby enhancing mRNA stability and expression (51). Meanwhile, IGF2BPs can recruit co-factors such as HuR and matrin 3 (MATR3) to prevent targeted mRNA from degradation, thereby enhancing the stability of IGF2BPs-mediated mRNA (48). In addition, IGF2BPs have been demonstrated to play a role in mRNA translation through modulating alternative splicing (52). Accumulating data has shown that IGF2BPs are abnormally expressed in many malignancies, which always exhibit oncogenic effects and correlate with the poor prognosis of cancer patients (53). Therefore, further studies are strongly necessary to understand the intricate regulatory mechanisms of IGF2BPs in tumorigenesis and development.

#### 2.2.3 The HNRNP family protein

The proteins of the Heterogeneous nuclear ribonucleoprotein (HNRNP) family form a structurally diverse group of RNA binding proteins (RBPs), and the different functions depend on HNRNP intracellular localization (54). The majority of HNRNPs have three fundamental structural domains: the RNA recognition motif (RRM), the Arginine-Glycine-Glycine (RGG), and the KH domain (KH) (55). Among them, HNRNPG recognizes RNAs through its RRM and RGG motifs and is capable of splicing m6A-modified RNAs (56). HNRNPC modulates translation efficiency in a stable and cell cycle-dependent manner through its interaction with multi-U tails present in the 3’- and 5’-UTRs of mRNA (57). HNRNPA2B1 interacts with specific RNA sequences and is involved in m6A modification through its RRMs. HNRNPA2B1 has been demonstrated to influence miRNA processing, facilitate nucleocytoplasmic trafficking, and stabilize m6A-modified mRNA (54, 58). Additionally, studies have demonstrated that the dysregulation of HNRNPs plays a critical role in tumor development and resistance (59). Through controlling alternative splicing and translation of RNAs, HNRNPs promote the diversity of aberrant proteomes associated with tumors and tumor immunity (59). Meanwhile, HNRNPs can also promote tumor-associated gene expression by regulating transcription factors, directing DNA binding, or promoting chromatin remodeling (58). These highlight the pivotal roles of HNRNPs in the biological mechanisms of tumorigenesis and development.

## 3 Interactions between ALKBH5 and reader proteins in tumors

**Figure 2:**
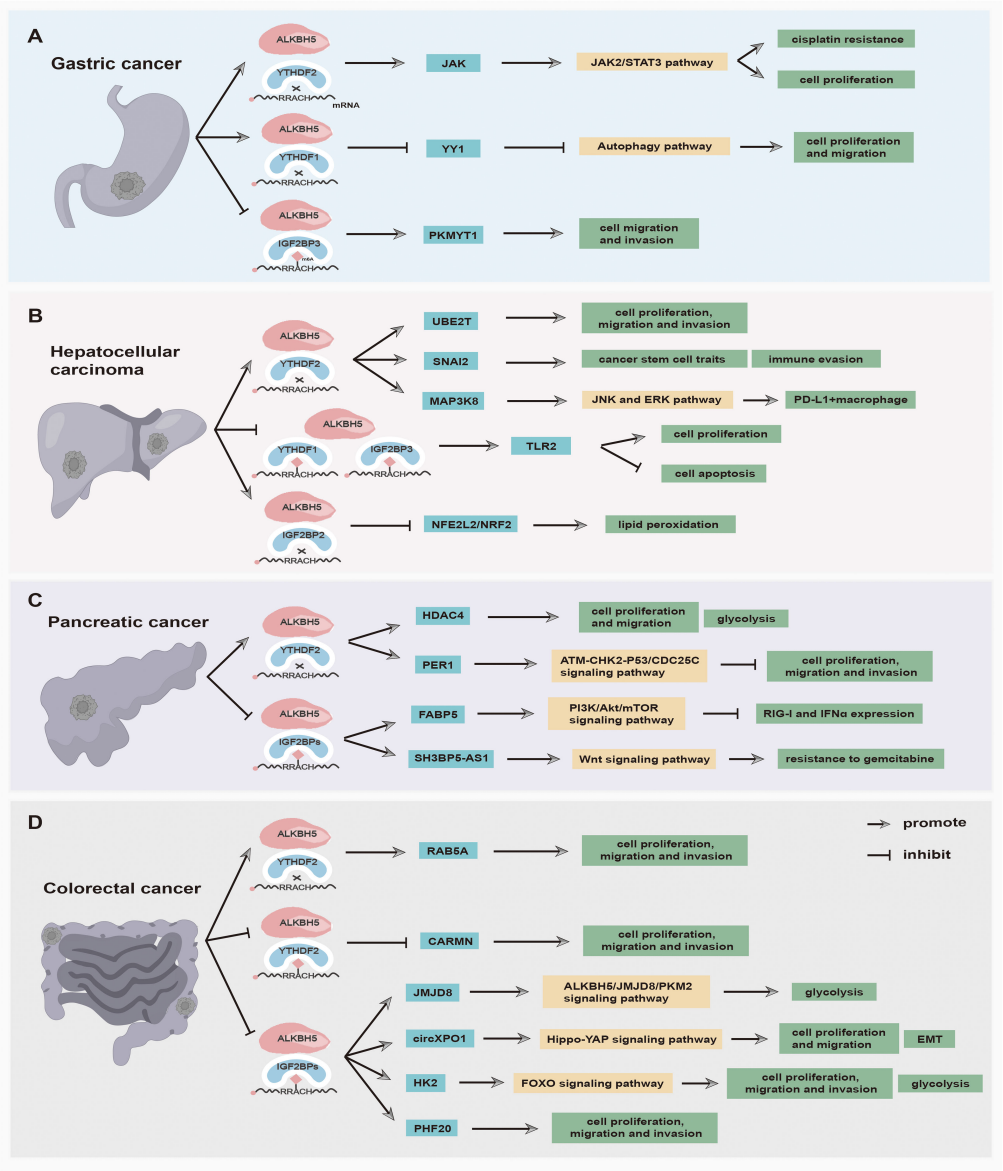
The main functions and synergistic mechanisms between ALKBH5 and reader proteins in digestive system tumors. **(A)** Gastric cancer, **(B)** Hepatocellular carcinoma, **(C)** Pancreatic cancer, and **(D)** Colorectal cancer.

Increasing evidence has suggested that the m6A demethylase ALKBH5 and reader proteins are aberrantly expressed in various tumors (25). The interactions between ALKBH5 and reader proteins are closely associated with tumorigenesis and development, making them potential novel targets for tumor prevention and treatment (**Table 1**). In the following section, the expression of ALKBH5 and reader proteins, as well as the synergistic mechanisms between ALKBH5 and reader proteins in tumors from different systems, is summarized and discussed. Among them, the synergistic mechanisms between ALKBH5 and reader proteins in digestive system tumors are illustrated in **Figure 2**.

**Table 1:** Interactions between ALKBH5 and reader proteins in human cancers.

Roles of ALKBH5	Cancer	Reader protein	Downstream targets	Molecular mechanisms	Target pathways	Functional effects	Ref.
Oncogene	Non-small cell lung cancer	YTHDF2	SAMD7, SOX2, MYC	Decreased YTHDF2-dependent mRNA decay	–	Proliferation, migration, colony formation	(35)
	Non-small cell lung cancer	YTHDF2	JAK2	Decreased YTHDF2-dependent mRNA decay	JAK2/p-STAT3 signaling pathway	Proliferation, TAMs recruitment, M2 macrophage polarization	(60)
	Non-small cell lung cancer	YTHDC2	CALML3-AS1	Decreased YTHDC2-dependent mRNA stabilization	–	Proliferation, migration	(61)
	Non-small cell lung cancer	IGF2BPs	CDKN1A, TIMP3	Decreased IGF2BPs-dependent mRNA stabilization	–	Proliferation, apoptosis	(62)
	Lung adenocarcinoma	YTHDF1	ENO1	Promotion in translation by YTHDF1	–	Glycolysis	(63)
	Head and neck squamous cell carcinoma	HNRNPC	DDX58	Decreased HNRNPC-dependent mRNA maturity	IKKϵ/TBK1/IRF3 signaling pathway	Inhibits RIG-I expression and IFNɑ production	(64)
	Colorectal cancer	YTHDF2	RAB5A	Decreased YTHDF2-dependent mRNA decay	–	Proliferation, migration, invasion	(65)
	Hepatocellular carcinoma	YTHDF2	UBE2T	Decreased YTHDF2-dependent mRNA decay	–	Proliferation, migration, invasion	(66)
	Hepatocellular carcinoma	YTHDF2	SNAI2	Decreased YTHDF2-dependent mRNA decay	–	Maintained cancer stem cell traits, immune evasion	(67)
	Hepatocellular carcinoma	YTHDF2	MAP3K8	Decreased YTHDF2-dependent mRNA decay	JNK and ERK signaling pathways	Proliferation, metastasis, macrophage recruitment	(68)
	Gastric cancer	YTHDF2	JAK	Decreased YTHDF2-dependent mRNA decay	JAK1/STAT3 signaling pathway	Proliferation, metastasis	(69)
	Pancreatic cancer	YTHDF2	HDAC4	Decreased YTHDF2-dependent mRNA decay	–	Glycolysis, proliferation, migration	(70)
	Pancreatic neuroendocrine neoplasms	IGF2BP2	FABP5	Increased IGF2BP2-dependent mRNA stabilization	PI3K/Akt/mTOR signaling pathway	Proliferation, migration, invasion, lipid metabolism	(71)
	Cervical cancer	YTHDF2	PAK5	Decreased YTHDF2-dependent mRNA decay	–	Proliferation, migration, invasion	(72)
	Cervical cancer	YTHDF2	circCCDC134	Decreased YTHDF2-dependent mRNA decay	–	Proliferation, metastasis	(73)
	Bladder cancer	YTHDF1/3	ITGA6	Promotion in translation by YTHDF1/3	–	Cell adhesion, proliferation, migration	(74)
	Epithelial ovarian cancer	YTHDF2	JAK2	Decreased YTHDF2-dependent mRNA decay	JAK2/STAT3 signaling pathway	Proliferation, cisplatin resistance	(75)
	Epithelial ovarian cancer	YTHDF2	ITGB1	Decreased YTHDF2-dependent mRNA decay	–	Migration, invasion	(76)
	Osteosarcoma	YTHDF2	PVT1	Decreased YTHDF2-dependent mRNA decay	–	Proliferation	(77)
	Multiple myeloma	YTHDF2	TRAF1	Decreased YTHDF2-dependent mRNA decay	NF-κB and MAPK signaling pathway	Proliferation	(78)
	Breast cancer	YTHDF2	LINC00115	Decreased YTHDF2-dependent mRNA decay	SETDB1/PLK3/HIF1α signaling pathway	Chemoresistance, metastasis	(79)
	Breast cancer	YTHDF2	GLUT4	Decreased YTHDF2-dependent mRNA decay	–	Glycolysis, drug resistance	(80)
	Intrahepatic cholangiocarcinoma	YTHDF2	PD-L1	Increased YTHDF2-dependent mRNA decay	–	Promoted PD-L1 expression and MDSCs infiltration	(81)
	Acute myeloid leukemia	YTHDF2	AXL	Decreased YTHDF2-dependent mRNA decay	PI3K/AKT/mTOR pathway	Proliferation, survival, stem cell self-renewal	(82)
	Glioma	YTHDF2	ZDDHC3	Decreased YTHDF2-dependent mRNA decay	–	Promoted immune escape	(83)
Cancer suppressor	Non-small cell lung cancer	YTHDF1/2/3	TGFβR2, SMAD3, SMAD6	Inhibition in translation by YTHDF1/3; decreased YTHDF2-dependent mRNA decay	TGF-β/SMAD signaling pathway	EMT, metastasis invasion	(34)
	Non-small cell lung cancer	YTHDF1/2/3	YAP, miR-107/LATS2	Competitive binding by YTHDF1 and YTHDF2; suppression of miR-107/LATS2-mediated YAP1 phosphorylation by HuR	–	EMT, proliferation, migration	(84)
	Non-small cell lung cancer	IGF2BP3	XBP1	Increased IGF2BP3-dependent mRNA stabilization	IL-6-JAK-STAT3 signaling pathway	proliferation, migration, invasion	(85)
	Hypopharyngeal squamous cell carcinoma	IGF2BP2	NFE2L2/NRF2	Decreased IGF2BP2-dependent mRNA stabilization	–	Ferroptosis, lipid peroxidation	(86)
	Hypopharyngeal squamous cell carcinoma	YTHDF1, IGF2BP2	TLR2	Promotion in translation by YTHDF1; increased IGF2BP2-dependent mRNA stabilization	–	Cell growth, proliferation, cell apoptosis	(87)
	Breast cancer	IGF2BP3	ESPL1	Decreased IGF2BP3-dependent mRNA stabilization	–	Proliferation, cell cycle	(88)
	Colorectal cancer	IGF2BPs	JMJD8	Increased IGF2BPs-dependent mRNA stabilization	ALKBH5/JMJD8/PKM2 signaling pathway	Glycolysis	(89)
	Colorectal cancer	IGF2BP2	circXPO1	Increased IGF2BP2-dependent mRNA stabilization	Hippo-YAP signaling pathway	Proliferation, migration, EMT	(90)
	Colorectal cancer	YTHDF2/3	CARMN	Increased YTHDF2-dependent mRNA decay	–	Proliferation, migration, invasion	(91)
	Colorectal cancer	IGF2BP3	PHF20	Increased IGF2BP3-dependent mRNA stabilization	–	Proliferation, migration, invasion	(92)
	Colorectal cancer	IGF2BP2	HK2	Increased IGF2BP2-dependent mRNA stabilization	FOXO signaling pathway	Glycolysis, proliferation, migration, invasion	(27)
	Cervical squamous cell carcinoma	IGF2BP1	SIRT3	Increased IFG2BP1-dependent mRNA stabilization	–	Fatty acid metabolism	(93)
	Pancreatic cancer	YTHDF2	PER1	Decreased YTHDF2-dependent mRNA decay	ATM-CHK2-P53/CDC25C signaling pathway	Proliferation, migration, invasion	(94)
	Pancreatic cancer	IGF2BP1	SH3BP5-AS1	Increased IFG2BP1-dependent mRNA stabilization	Wnt signaling pathway	Resistance to gemcitabine	(95)
	Hepatocellular carcinoma	IGF2BP1	LYPD1	Increased IFG2BP1-dependent mRNA stabilization	–	Proliferation, migration, invasion	(96)
	Hepatocellular carcinoma	IGF2BP1	PAQR4	Increased IGF2BP1-dependent mRNA stabilization	PI3K/AKT signaling pathway	Proliferation, migration, invasion	(26)
	Hepatocellular carcinoma	IGF2BP1	LINC02551	Decreased IGF2BP1-dependent mRNA stabilization	–	Proliferation, migration, invasion	(97)
	Gastric cancer	IGF2BP3	PKMYT1	Increased IGF2BP3-dependent mRNA stabilization	–	Migration, invasion	(98)
	Gastric cancer	YTHDF1	YY1	Inhibition in translation by YTHDF1	Autophagy	Proliferation, migration	(99)
	Osteosarcoma	YTHDF2	SOCS3	Increased YTHDF2-dependent mRNA decay	STAT3 signaling pathway	Proliferation, cell apoptosis and cycle arrest	(100)
	Osteosarcoma	YTHDF1/2	miR-181b-1, YAP	Increased YTHDF2-dependent mRNA decay; promotion in translation by YTHDF1	**-**	Proliferation, migration, invasion, cell apoptosis	(101)

### 3.1 Interactions between ALKBH5 and reader proteins in respiratory system tumors

#### 3.1.1 Lung cancer

Lung cancer is a highly prevalent and lethal malignancy in the world (102). In histopathology, lung cancer can be classified into non-small lung cancer (NSCLC) and small cell lung cancer (SCLC) (102). About 10% of NSCLC cases exhibit simultaneous KRAS mutations and LKB1 tumor-suppressor gene (KL) loss (103, 104). Compared with general NSCLC cells, NSCLC cells with KL exhibit a higher propensity for cellular metastasis and drug resistance (104). Increasing evidence has suggested that ALKBH5 acts as an oncogene and is abnormally expressed in NSCLC. The cross-talks between ALKBH5 and reader proteins are significantly associated with tumorigenesis. Zhang et al. (35) found that the high expression level of ALKBH5 is associated with poor survival for KL patients. The loss of LKB1 promotes DNA hypermethylation in the single putative transcriptional repressor, known as CTCF (CCCTC-binding factor), peak region in ALKBH5, thus upregulating the expression of ALKBH5 in KRAS mutant cells and facilitating histone modification (35). In the mechanism, m6A modification of SAMD7, SOX2, and MYC is demethylated by ALKBH5 and subsequently recognized by YTHDF2, resulting in upregulated expression of SAMD7, SOX2, and MYC, which strengthen LKB1-regulated cell proliferation, colony formation, and migration of KRAS-mutated lung cancer cells (35). In NSCLC, CDKN1A (p21) and TIMP3, which act as tumor suppressor genes, can be demethylated by overexpressed ALKBH5 and recognized by IGF2BPs, promoting the apoptosis, proliferation, and tumorigenesis of NSCLC cell *in vivo* and *in vitro* (62). In addition, the lncRNA CALML3 antisense RNA 1 (CALML3-AS1) is upregulated expression in NSCLC and positively correlated with m6A demethylation ALKBH5, whereas it is destabilized by YTHDC2 at the post-transcription level. The in-depth study demonstrates that CALML3-AS1 can inhibit BTNL9 transcription and expression through the recruitment of EZH2, giving rise to the counteraction of CALML3-AS1-mediated antitumor effects on NSCLC cells (61). Hua et al. have demonstrated that ALKBH5/YTHDF2-mediated m6A modification can upregulate the expression of JAK2 at the post-transcriptional level to facilitate NSCLC progression. Meanwhile, ALKBH5 can recruit PD-L1-positive tumor-associated macrophages (TAMs) and promote M2 macrophage polarization by inducing the secretion of CCL2 and CXCL10, ultimately accelerating the progression of NSCLC (60). The above findings suggest that targeting ALKBH5 may be a promising strategy for facilitating the prediction of clinical response to anti-PD-L1 immunotherapy. In addition, Ma et al. (63) have discovered that low-level ALKBH5 upregulates ENO1 expression by enhancing the YTHDF1-mediated translation of m6A-modified ENO1 mRNA, thus stimulating glycolysis and tumorigenesis of Lung adenocarcinoma (LUAD). Moreover, the downregulation of ALKBH5 promotes TGF-β-induced epithelial-mesenchymal transition (EMT) and invasion of NSCLC cells (34). Among them, TGFβR2 and SMAD3 are stabilized by ALKBH5/YTHDF1/3-mediated m6A methylation after transcription, while m6A demethylation caused by ALKBH5 downregulates SMAD6 in an m6A-YTHDF2-dependent manner (34). Additionally, ALKBH5 can act as a tumor suppressor to impede NSCLC progression by downregulating YTHDF1/2/3-mediated YAP expression and inhibiting miR-107/LATS2-mediated YAP activity (84). Furthermore, the ALKBH5 depletion increases m6A modification in the 3’UTR of XBP1 mRNA, and enhances its stabilization in an IGF2BP3-dependent way, activating the IL-6-JAK-STAT3 signaling pathway to promote the proliferation, migration, and invasion of NSCLC cells (85). These studies indicate that targeting ALKBH5 and reader proteins is a promising strategy for enhancing the diagnosis and therapy of lung cancer.

#### 3.1.2 Head and neck squamous cell carcinoma

Head and neck squamous cell carcinoma (HNSCC) is the sixth most common cancer worldwide with an incidence of approximately 700,000 confirmed cases every year, including thyroid, nasopharyngeal, hypopharyngeal squamous cell (HPSCC), and oral carcinoma (105). Although conventional treatment modalities, such as surgical intervention, chemotherapy, and radiotherapy, have significantly enhanced the prognosis of patients with HNSCC, the overall survival rate still remains low (106). The deep investigation of the molecular mechanisms underlying the cross-talks between ALKBH5 and reader proteins has the potential to improve targeted therapy for HNSCC. Jin et al. (64) discovered that ALKBH5 plays an oncogene role in HNSCC. The upregulated ALKBH5 demethylates m6A modification of DDX58 mRNA, which suppresses the maturity of DDX58 mRNA by preventing HNRNPC binding to its m6A sites, thus promoting HNSCC progression through the IKKϵ/TBK1/IRF3 pathway (64). In addition, ALKBH5 can also function as a suppressor gene in HPSCC. The downregulated ALKBH5 increases m6A methylation of TLR2 mRNA at 3’UTR and enhances TLR2 stability and translation respectively by IGF2BP2 and YTHDF1 dependent way to promote HPSCC cell proliferation (87). Furthermore, inducing ferroptosis in cancer cells can effectively overcome cisplatin resistance (107). However, the highly expressed ALKBH5 impairs NFE2L2/NRF2mRNA expression in an m6A-IGF2BP2-dependent fashion at the post-transcriptional level, inhibiting the resistance of HPSCC cells to ferroptosis (86).

Currently, the roles of ALKBH5 interacting with reader proteins in HNSCC are gradually being uncovered. Further studies should be investigated to provide insights into the interactions between ALKBH5 and reader proteins that regulate HNSCC, as well as to identify relevant diagnostic and therapeutic targets.

### 3.2 Interactions between ALKBH5 and reader proteins in digestive system tumors

#### 3.2.1 Gastric cancer

The prevalence of gastric cancer (GC) among young adults has been steadily increasing in both low-risk and high-risk countries in recent years (108). Patients of GC are usually diagnosed at an advanced stage, thereby having a dismal prognosis and low survival rate (109). At the cellular level, epigenetic alterations in GC cells are closely related to their invasive and metastatic behavior (110). Therefore, investigating RNA modifications in GC invasion and metastasis holds promise for uncovering novel therapeutic strategies. In GC samples, ALKBH5 can function as a tumor suppressor gene. Hu et al. (98) have demonstrated that the ALKBH5 downregulation is associated with metastasis of clinically distant tumors and lymph nodes. The low ALKBH5 increases the expression of its downstream target PKMYT1 via the ALKBH5-PKMYT1-IGF2BP3 axis to promote the invasion and migration of GC (98). In the mechanism, ALKBH5 removes m6A modification in PKMYT1 3’UTR and enhances its stabilization via an m6A-IGF2BP3-dependent manner, thus promoting and facilitating tumorigenesis (98). The zinc-finger transcription factor Yin-Yang 1 (YY1) is identified to act as a crucial transcriptional activator of cancer autophagy-related genes (99). ALKBH5-mediated YY1 demethylation decreases the translation of YY1 transcript through the YTHDF1-dependent way, which then suppresses the transactivation of ATG4B, suggesting that ALKBH5 inhibits the GC progression via regulating the ALKBH5/YY1/ATG4B axis (99). Additionally, ALKBH5 can play an oncogenic role in GC as well. LINC00659 reduces the m6A abundance of JAK1 mRNA by enhancing the demethylase activity of ALKBH5, resulting in the upregulation of JAK1 mRNA through inhibiting JAK mRNA degradation mediated by YTHDF2 protein (69). The high level of JAK1 ultimately activates the JAK1/STAT3 pathway to facilitate GC tumorigenesis and development (69). Considering the dual function of ALKBH5 in GC, it is meaningful to further explore the underlying interaction between ALKBH5 and reader proteins in the occurrence and development of GC.

#### 3.2.2 Hepatocellular carcinoma

Hepatocellular carcinoma (HCC) primarily occurs in individuals with chronic liver disease at the cirrhosis stage (111). When the disease progresses to an advanced stage, many HCC patients cannot undergo surgical transplantation or resection, while some patients with molecular targeted therapy still have poor curative effects due to HCC recurrence and cell drug resistance (112). A study has revealed that ALKBH5 plays a suppressor role in HCC and promotes the overall survival of HCC patients (96). Indeed, the upregulated ALKBH5 post-transcriptionally weakens LYPD1 expression in an m^6^A-IGF2BP-dependent manner, inhibiting HCC cell proliferation and invasion (96). The high level of ALKBH5 also impairs m6A modification in PAQR4 mRNA, which then hampers its recognition by IGF2BP1, thus weakening the expression of PAQR4 and inhibiting the activation of the PI3K/AKT pathway to impede the occurrence and development of HCC (26). Moreover, the lncRNA LINC02551 is downregulated by the high level of ALKBH5 and suppresses recognition mediated by IGF2BP1 at the post-transcription level (97). The downregulation of LINC02551 promotes TRIM27-induced DDX24 degradation, resulting in the suppression of HCC growth (97). Furthermore, ALKBH5 can also act as an oncogene in HCC. LncRNA cancer susceptibility candidate 11 (CASC11), an oncogene, is overexpressed in HCC tissues and cells and decreases the m6A level of UBE2T via recruiting ALKBH5 (66). CASC11 can also inhibit the interaction between UBE2T mRNA and YTHDF2, aiming to impede the degradation of UBE2T mRNA to accelerate HCC progression (66). Besides, Hepatitis B viral (HBV) persistent infection plays a critical role in HCC tumorigenesis (113). Meng et al. (67) found that ALKBH5 overexpression demethylates the m6A modification in the 3’ UTR of the oncogenic gene SNAI2 to prevent recognition mediated by YTHDF2, thus stabilizing SNAI2 transcripts to maintain cancer stem cell traits in HBV-positive HCC. Additionally, the ALKBH5/SNAI2 axis accelerates tumor immune evasion through increasing the expression of the ligand of immune checkpoint CD155 (67). Ultimately, You et al. (68) found that a high level of ALKBH5 impels the stability of MAP3K8 mRNA in an m6A-YTHDF2-dependent manner and upregulates MAP3K8 expression to promote the activation of IL-8 via the JNK and ERK pathways, thus recruiting PD-L1+ macrophages to participate in shaping the immunosuppressant microenvironment of HCC. This finding suggests that targeting ALKBH5 may enhance the outcomes of anti-PD-L1 immunotherapy in HCC.

#### 3.2.3 Pancreatic cancer

Pancreatic cancer (PC) is a deadly malignancy with limited efficacy of chemotherapy, and more than 90% of pancreatic tumors arise from the ductal epithelium (114). Early-stage PC often exhibits micro-metastasis, which contributes to a poor prognosis and high mortality rate (114). Another pancreatic tumor, pancreatic neuroendocrine neoplasm (pNEN), is derived from the islet cells of the pancreas, which have had an increasing incidence in recent years (115). In recent years, proteins that function as erasers and readers of m6A modifications have been shown to play critical roles in PCs. Guo et al. (94) demonstrated that the upregulated ALKBH5 suppresses the proliferation, migration, and invasion of PC cells via the ATM-CHK20P53/CDC25C axis *in vitro* and *in vivo*. ALKBH5 downregulates m6A levels of PER1 mRNA, and the m6A alteration of PER1 mRNA is identified by YTHDF2, which then inhibits degradation to increase PER1 production, thus reactivating ATM-CHK2-P53/CDC25C signaling to suppress PC growth (94). In addition, the low-expression level of ALKBH5 increases the m6A modification in SH3BP5-AS1 mRNA, which is then read and stabilized by IGF2BP1, activating the Wnt signaling pathway by sponging miR-139-5p to increase CTBP1 expression, thereby enhancing the gemcitabine resistance of PC (95). Furthermore, Li et al. (70) found that the high level of ALKBH5 regulates hypoxia-induced PC glycolytic metabolism via the ALKBH5/HDAC4/HIF1α positive feedback loop. Under hypoxic conditions, the hypoxia-indued HDAC4 enhances HIF1α protein stability, and overexpressed HIF1a promotes transcription of ALKBH5, which in turn decreases m6A modification in HDAC4 mRNA, finally accelerating HDAC4 production via an m6A-YTHDF2-dependent manner to promote glycolytic metabolism and migration of PC cells (70). Chen et al. (71) discovered that ALKBH5 is overexpressed in pNENs and plays a promoter role in tumor growth and lipid metabolism. In the mechanism, ALKBH5-driven 5’UTR m6A demethylation enhances the expression of FABP5 in an m6A-IGF2BP2 dependent way, and ALKBH5 can activate the PI3K/Akt/mTOR signaling pathway, which collectively enhances lipid metabolism and proliferation of pNENs cells (71).

#### 3.2.4 Colorectal cancer

Colorectal cancer (CRC) is the third most common cancer with a high mortality rate (116). Clinical data has revealed that numerous risk factors, such as smoking, elevated BMI, and excessive alcohol consumption, are associated with an increased CRC incidence. The overexpression of ALKBH5 is associated with unfavorable prognosis for CRC patients and promotes the proliferation, migration, and invasion of CRC cells *in vivo* and *in vitro*. In-depth studies found that upregulated expression of ALKBH5 can lead to stability enhancement of RAB5A, which impedes YTHDF2-mediated RAB5A mRNA decay and increases RAB5A expression, ultimately influencing the tumorigenicity of CRC (65). Interestingly, it has been reported that ALKBH5 can play a crucial tumor suppressive role in CRC as well. ALKBH5 transcription is reduced by histone deacetylase 2-mediated H3K27 deacetylation and accelerates CRC occurrence and development (89). The downregulated ALKBH5 results in increased m6A modification in JMJD8 mRNA, which is then read and stabilized by IGF2BPs, promoting glycolysis through the ALKBH5/JMJD8/PKM2 signaling axis (89). CircXPO1 is upregulated in CRC tissues and cells, which is positively related to the growth, EMT, and metastasis of CRC cells. CircXPO1 is stabilized depending on ALKBH5/IGF2BP2-mediated m6A methylation after transcription and reduces the mRNA stability of WWC2 by interacting with FMRP, which consequently results in Hippo-YAP pathway activation to facilitate CRC progression (90). According to Liu’s study, in CRC cells, mutant P53 can bind to the ALKBH5 promoter to impede ALKBH5 transcription, enhancing m6A methylation levels on lncRNA CARMN. The m6A methylated lncRNA CARMN is then bound and degraded by reader proteins YTHDF2/YTHDF3 in an m6A-dependent manner, hence downregulating CARMN expression (91). Elevated ALKBH5 levels can inhibit CRC cells by destabilizing PHF20 in an m6A-IGF2BP3-dependent way (92). The in-depth study has found that ALKBH5 removes the m6A modification on PHF20 mRNA and decreases PHF20 mRNA stabilization by weakening the function of IGF2BP3 (92). More interestingly, the high expression of ALKBH5 can also inhibit CRC cells in a high-fat environment (27). In terms of metabolism, HK2 is a key enzyme for glucose glycolysis. The FTO-ALKBH5/IGF2BP2-m6A axis mediates the stabilization of HK2 mRNA, leading to the downregulation of HK2 expression, which restrains the FOXO signaling pathway to suppress CRC progression (27). This implies that the FTO-ALKBH5/IGF2BP2-m6A axis may be a potential therapeutic target for CRC.

Overall, the synergistic mechanisms between ALKBH5 and reader proteins in the digestive system tumors are summarized, while the regulatory factors may have opposing effects on different tumors, as well as within the same tumor in different patients. For instance, while ALKBH5 acts as a tumor suppressor in HCC, it may play an oncogene role in GC, which requires further investigation. Additionally, the research of interaction networks among lncRNA, mRNA, and methylation modifications in digestive system tumors is currently insufficient and is expected to become a new research hotspot in the future.

### 3.3 Interactions between ALKBH5 and reader proteins in genitourinary system tumors

#### 3.3.1 Cervical cancer

Cervical cancer (CC) is the fourth most prevalent malignant tumor among women in the world, resulting in over 250,000 deaths, and approximately 75% of cases are attributed to Cervical squamous cell carcinoma (CESC) (117). Clinical evidence shows that advancing age predicts a poor prognosis for CC, which will have an undesirable impact on treatment strategies and overall survival rates (118). Infection with high-risk human papillomavirus (HPV) is closely associated with CC development (119). Huo et al. (72) found that Human papillomavirus (HPV) E7 can promote ALKBH5 expression by E2F1-mediated H3K27Ac and H3K4Me3 activation histone modifications as well as DDX3-mediated post-translation modification. Whereafter, the overexpressed ALKBH5 weakens m6A methylations in the 3’UTR of PAK5 transcript, inhibiting PAK5 protein degradation via an m6A-YTHDF2-dependent way, thereby facilitating the occurrence and development of CC (72). Furthermore, the upregulated ALKBH5-mediated circCCDC134 demethylation raised the stability of circCCDC134 in the YTHDF2-dependent manner (73). CircCCDC134 overexpression facilitates the recruitment of p65 in the nucleus and acts as a miR-503-5p sponge to modulate the expression of MYB in the cytoplasm, ultimately stimulating HIF1A transcription and promoting CC growth and metastasis (73). Currently, studies have demonstrated that the metabolic reprogramming of CESC, regulated by m6A regulators, can remodel the tumor microenvironment and impact tumor growth (120). Indeed, ALKBH5 can be a cancer suppressor by modulating the lipid metabolism of CESC. To be specific, the evaluation of the correlation between ALKBH5 levels and survival in CESC patients has revealed that increased ALKBH5 can regulate the SIRT3/ACC1 axis to inhibit CESC lipid metabolism and improve patient prognosis (93). The high-level ALKBH5 reduces m6A methylation of SIRT3 mRNA at 3’UTR, leading to decreased stability and levels of SIRT3 mRNA mediated by IGF2BP1 protein. The SIRT3 repression results in reduced deacetylation of ACC1 expression, ultimately inhibiting CESC lipid metabolism (93). Therefore, aiming to have novel options for future treatment of CESC, it is essential to deeply investigate the synergistic mechanisms between ALKBH5 and reader proteins underlying the CESC metabolic reprogramming.

#### 3.3.2 Bladder cancer

Bladder cancer (BLCA) is a common malignant tumor in the genitourinary system, which is classified as non-muscle-invasive BLCA (NMIBLCA) and muscle-invasive BLCA (MIBLCA) (116). NMIBLCA, accounting for approximately 75% of BLCA, frequently recurs. In 10–30% of patients, NMIBLCA can progress to MIBLCA. This progression is associated with a reduced survival rate and frequent distant metastasis (121). Due to its high heterogeneity and recurrence rate, BLCA remains a major cause of cancer-related morbidity and mortality (122). Hence, it is necessary to explore the underlying molecular mechanisms of BLCA tumorigenesis and development. Jin et al. (74) have discovered that the upregulation of ITGA6 is associated with lower survival rates in BLCA patients. In this mechanism, high-level METTL3 and low-level ALKBH5 contribute to enriched m6A modification in ITGA6 transcripts, promoting the translation of ITGA6 mRNA mediated by YTHDF1 and YTHDF3 proteins, which results in facilitating BLCA progression (74). At present, there is insufficient research on the interaction of ALKBH5 with reader proteins in modulating tumorigenesis and the development of BLCA, urgently requiring additional investigation.

#### 3.3.3 Ovarian cancer

Ovarian cancer (OC) is the seventh most common cancer worldwide and the fifth leading cause of cancer‐related death in women (123). OC is classified into three categories according to the type of cell source: epithelial, stromal, and germ cell tumors (124). Among them, epithelial ovarian cancer (EOC) accounts for 90%–95% of malignant ovarian tumors. Due to its asymptomatic progression and early peritoneal dissemination, OC is often diagnosed in its terminal stage, resulting in a low survival rate of OC patients (124). Patients diagnosed with advanced EOC often undergo platinum-based chemotherapy, but approximately 75% of these patients will develop platinum resistance within the initial five treatment years (123), suggesting that new treatment strategies are needed. Nie et al. (75) have demonstrated that the ALKBH5-HOXA10 loop is positively regulated in cisplatin-resistant EOC and promotes cisplatin resistance of EOC cells *in vivo* and *in vitro*. HOXA10 is the upstream transcription factor of ALKBH5 and forms a loop with ALKBH5, which upregulates ALKBH5 to weaken m6A abundance in JAK2 mRNA, inhibiting JAK2 mRNA degradation mediated by YTHDF2 (75). The high-level JAK2 activates the JAK2/STAT3 signaling pathway to eventually induce EOC resistance to cisplatin. Furthermore, the upregulated HIF1α promotes ALKBH5 overexpression, which can increase tumor-related lymphogenesis and lymph node (LN) metastasis of EOC *in vivo* and *in vivo* (76). In this mechanism, ITGB1, upregulated by ALKBH5/YTHDF2-mediated regulation, can lead to phosphorylation of the focal adhesion kinase (FAK) and Src proto-oncogene proteins, thus promoting LN metastasis (76).

Above all, the interaction mechanisms between ALKBH5 and reader proteins in genitourinary system tumors are summarized, providing novel insights for tumor treatment. In OC, ALKBH5 and reader proteins play positive roles in the tumorigenesis and development of EOC. However, it still needs to be considered whether the effect of ALKBH5 and reader proteins is consistent across different categories of OC. Furthermore, it remains unclear whether ALKBH5 can interact with other reader proteins, such as YTHDCs, and HNRNPs, to modulate tumor progression in the genitourinary system, which needs further investigation.

### 3.4 Interactions between ALKBH5 and reader proteins in other system tumors

#### 3.4.1 Osteosarcoma

Osteosarcoma (OS), originating from primitive bone-forming mesenchymal cells, is the most common bone malignancy in children and adolescents with a secondary peak in occurrence among individuals over 50 years of age (125). There is increasing evidence that OS may be related to cancer stem cells (CSCs), DNA repair-associated gene defects, tumor inhibition pathways, and genetic alterations (126). Hence, an urgent need is to deeply understand the underlying molecular mechanisms of OS occurrence and development, hoping to develop new therapeutic strategies to improve the prognosis of patients with OS. The highly expressed ALKBH5 reduces the m6A modification of PVT1 to suppress the YTHDF2-mediated m6A-dependent degradation, leading to PVT1 overexpression, which results in the promotion of OS cell proliferation and tumor growth (77). Interestingly, Yang et al. (100) have demonstrated that low-level ALKBH5 is associated with poor survival rates in OS patients, suggesting the tumor suppressor role of ALKBH5 in OS. In the mechanism, m6A abundance of SOCS3 mRNA is augmented by downregulated ALKBH5 and recognized by YTHDF2, leading to the degradation of SOCS3 that inactivates the STAT3 pathway, which promotes the tumorigenicity of OS (100). Additionally, Ye et al. (101) found that ALKBH5 silence enhances m6A methylations of pre-miR-181b-1 and YAP-mRNA exerting oncogenic functions in OS. YTHDF2 recognizes and reads m6A sites in pre-miR-181b-1 to mediate its degradation, while YTHDF1 binds to m6A-modified sites in YAP-mRNA to promote its translation, hence separately decreasing mature miR-181b-5p expression and increasing YAP expression to facilitate the OS progression (101). Currently, the roles of ALKBH5 and reader proteins in OS, as well as their synergistic mechanisms, are still controversial. Thus, there is an urgent need for additional evidence.

#### 3.4.2 Breast cancer

Breast cancer (BC) is a common malignant tumor and its incidence rate has shown an overall increasing trend in the past decade (127). Despite advancements in understanding and treating breast cancer, nearly 30% of patients experience recurrence or metastasis due to the lack of effective treatment or prevention strategies, which is the main reason for BC-related mortality (128). Numerous studies have shown the existence of m6A regulator disorder in BC, suggesting that m6A could be a potential therapeutic target for BC (129). Luo et al. (79) discovered that LINC00115 promotes chemo-resistant BC stem-like cell (BSCS) stemness and metastasis via the SETDB1/PLK3/HIF1α signaling pathway. To be more specific, LINC00115 acts as a scaffold lncRNA that connects SETDB1 and PLK3, resulting in enhanced SETDB1 methylation of PLK3 at both K106 and K200 in drug-resistant BCSC (79). The methylation of PLK3 decreases its phosphorylation of HIF1α, thereby increasing HIF1α stability. In turn, HIF1α upregulates ALKBH5 to post-transcriptionally enhance LINC00115 expression in an m6A-YTHDF2-dependent fashion, which eventually forms a positive feedback loop to provoke BCSC phenotypes, enhances chemoresistance and metastasis in triple-negative BC (79). In addition, Liu et al. (80) found that highly expressed ALKBH5 decreases m6A abundance in the 3’-UTR of GLUT4 mRNA and suppresses the degradation of GLUT4 transcripts by YTHDF2-mediated m6A reading, thus promoting glycolysis and resistance to HER2-targeted therapy in BC. Interestingly, ALKBH5 can also function as a tumor suppressor in BC. Zhang et al. (88) found that targeted inhibition of BRD4 can significantly upregulate the level of ALKBH5 protein by suppressing its ubiquitination and degradation processes. Subsequently, the overexpressed ALKBH5 demethylates ESPL1 mRNA and decreases the binding between ESPL1 mRNA and IGF2BP3, which facilitates the degradation of ESPL1 and ultimately inhibits the progression of BC (88). Hence, targeting ALKBH5 and reader proteins may improve the antitumor therapy of BC.

#### 3.4.3 Intrahepatic cholangiocarcinoma

About 20%-30% of patients with Intrahepatic cholangiocarcinoma (ICC) are eligible for resection, but the prognosis remains poor (130). Xin et al. (81) found that the deficient ALKBH5 enriches m6A modification in the 3’ UTR region of PD-L1 mRNA, promoting YTHDF2 binding to the 3’ UTR region of PD-L1 and facilitating PD-L1 mRNA degradation. The PD-L1 downregulation inhibits the cytotoxicity of T cells and enhances ICC growth, revealing that ALKBH5 can serve as a potential immunotherapy target in ICC.

#### 3.4.4 Multiple myeloma

Multiple myeloma (MM) is a hematological malignancy characterized by uncontrolled proliferation of monoclonal plasma cells in the bone marrow (BM) (131). The progression of MM is related to the accumulation of acquired genetic events and additional epigenetic alterations (132). The ALKBH5 overexpression plays an oncogenic role in MM, which can decrease m^6^A abundance in the 3’-UTR of TRAF1 transcripts (78). The stability of TRAF1 mRNA is then enhanced by YTHDF2 in an m6A-mediated manner, resulting in the activation of NF-κB and MAPK signaling pathways to accelerate MM cell growth and survival (78).

#### 3.4.5 Acute myeloid leukemia

Acute myeloid leukemia (AML) is a highly aggressive and fatal hematologic malignancy characterized by uncontrolled growth of immature myeloid cells (133). The development of AML is related to the accumulation of acquired genetic and epigenetic changes in hematopoietic stem/progenitor cells (HSPCs) (134). Wang et al. (82) have demonstrated that high-expressed ALKBH5 is essential for maintaining the function of human AML leukemia stem cells (LSCs) and is associated with poor prognosis of patients with AML. KDM4C upregulates ALKBH5 expression by increasing chromatin accessibility at the ALKBH5 locus, as well as reducing H3K9me3 levels and promoting recruitment of MYB and Pol II (82). In addition, ALKBH5 post-transcriptionally enhances receptor tyrosine kinase AXL expression via an m6A-YTHDF2-dependent fashion, activating the PI3K/AKT signaling pathway and facilitating AML progression (82).

#### 3.4.6 Glioma

Glioma, a highly heterogeneous tumor originating from the supportive glial cells of the central nervous system (CNS), is the most diagnosed primary tumor with rapid progression, inevitable recurrence, and poor abysmal prognosis (135). Although the approach of surgical resection combined with chemotherapy and radiotherapy has been used, the median overall survival for GBM patients is currently estimated to range from 12 to 16 months, urgently needing novel treatment methods (136). Tang et al. (83) showed that ALKBH5 silencing inhibits the growth of glioma allografts, rescues the antitumoral immune response, and increases cytotoxic lymphocyte infiltration as well as proinflammatory cytokines in cerebrospinal fluid (CSF). Indeed, low-level ALKBH5 increases m6A methylations in the 3’UTR of the ZDDHC3 transcript, suppressing ZDDHC3 expression mediated by YTHDF2, which then promotes the degradation of PD-L1 to decrease PD-L1 expression and ultimately inhibits immune escape (83). Additionally, IOX1, an inhibitor of ALKBH5, has been shown to enhance the effectiveness of anti-PD-1 therapy in a mouse model of Glioma (83). This suggests that the combination of anti-PD-1 therapy and ALKBH5 inhibition may represent a promising treatment strategy for glioma.

## 4 Potential applications of targeting ALKBH5 and reader proteins in antitumor drug development

The roles of both ALKBH5 and reader proteins in promoting tumorigenesis and development across many tumor types have established them as emerging novel therapeutic targets (137). Here, the potential applications of targeting ALKBH5 and reader proteins are summarized and discussed.

### 4.1 Inhibitors of ALKBH5 and reader proteins

**Figure 3:**
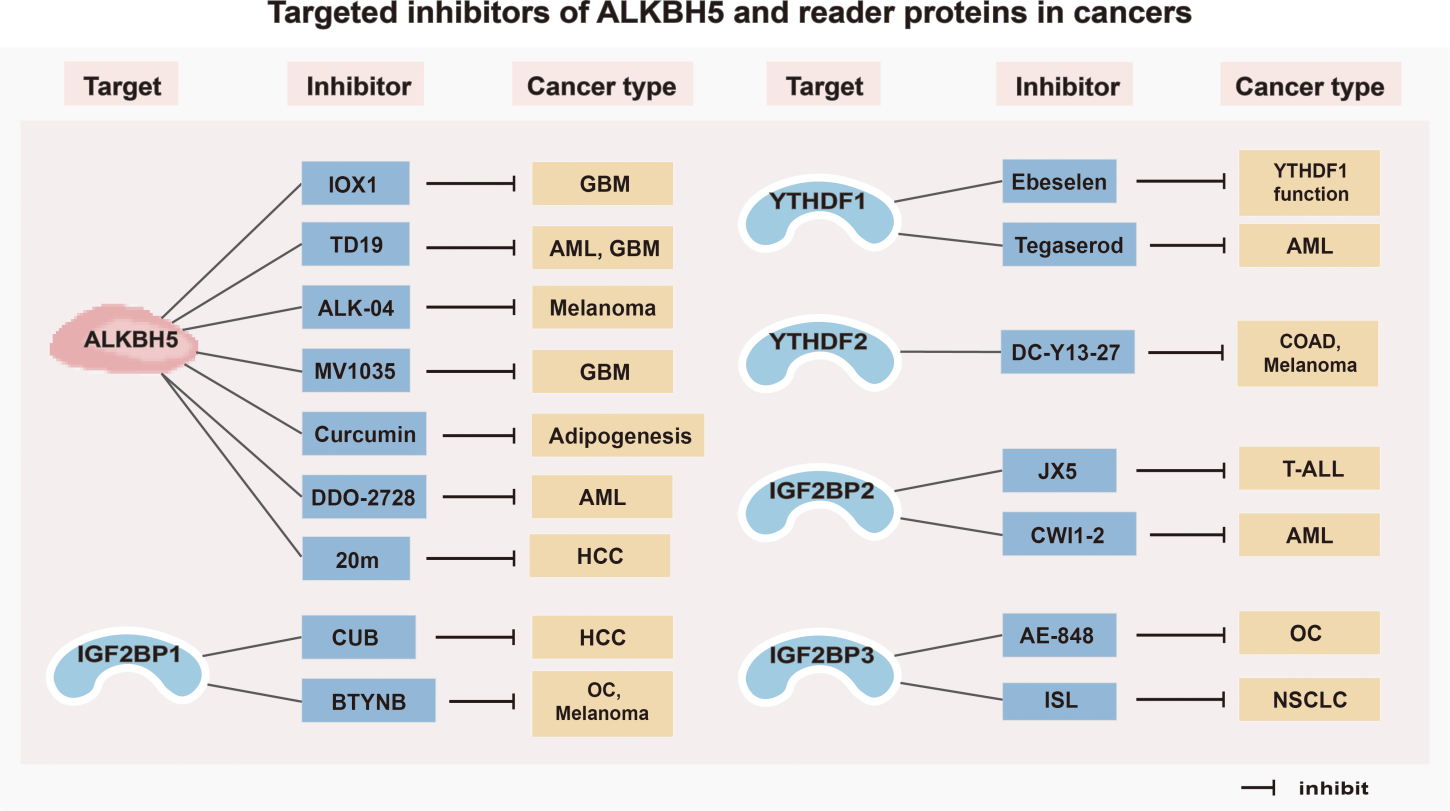
Targeted inhibitors of ALKBH5 and reader proteins in cancers. *GBM*, Glioblastoma; *AML*, Acute myeloid leukemia; *HCC*, Hepatocellular carcinoma; *OC*, Ovarian cancer; *COAD*, Colorectal adenocarcinoma; *T-ALL*, T-cell acute lymphoblastic leukemia; *NSCLC*, Non-small cell lung cancer.

With an increasing understanding of the crystal structures of both ALKBH5 and reader proteins, and their molecular mechanisms acting in tumors, various inhibitors have been developed and continuously optimized. These inhibitors have been demonstrated therapeutic effects in numerous tumors, providing a strong foundation for their future application. The potential impacts of ALKBH5 and reader protein inhibitors are detailed in **Table 2** and **Figure 3**.

**Table 2:** Effects of inhibitors of ALKBH5 and reader proteins.

Targets	Inhibitors	Cancer types	Function	Ref.
ALKBH5	2-{[1-hydroxy-2-oxo-2-phenylethyl] sulfanyl} acetic acid, 4- {[furan-2- yl] methyl} amino-1,2-diazinane-3,6- dione	Leukemia	Inhibits the proliferation of leukemia cells (HL-60, CCRF-CEM, and K562)	(138)
	ALK-04	Melanoma	Inhibits the infiltration of Treg cells and MDSCs; enhances the anti-PD-1 therapy	(139)
	MV1035	GBM	Inhibits the migration and invasion in U87 GBM cell lines	(140)
	DDO-2728	AML	Impedes cell cycle progression	(141)
	TD19	AML, GBM	Enhances anti-cancer efficacy	(142)
	Curcumin	–	Inhibits adipogenesis	(143)
	IOX1	GBM	Inhibits ALKBH5 activity via a cofactor 2- oxoglutarate oxygenase competitive manner	(83)
	5-hydroxy-1-(3- (trifluoromethyl) phenyl)-1 H-pyrazole- 3-carboxylic acid (20 m)	HCC	Inhibits ALKBH5 in HepG2 cells and increases m6A levels	(144)
YTHDF1	Ebeselen	–	Disrupts the interaction between YTHDF1 and its target mRNA	(145)
	Tegaserod	AML	Inhibits the viability of patient-derived AML cells	(146)
YTHDF2	DC-Y13-27	COAD, Melanoma	Inhibits the increase of ionizing radiation (IR) induced MDSC; enhances the anti-PD-L1 therapy	(147)
IGF2BP1	BTYNB	Melanoma, OC	Inhibits the proliferation of Melanoma and OC cells	(148)
	CuB	HCC	Induces cell apoptosis and enhances immune response	(149)
IGF2BP2	JX5	T-ALL	Inhibits the proliferation of T-ALL cells	(150)
	CWI1-2	AML	shows anti-leukemia effects in vitro and in vivo	(151)
IGF2BP3	AE-848	OC	Inhibits the growth and progression of OC cells	(152)
	Isoliquiritigenin (ISL)	NSCLC	Inhibit the proliferation, migration, and invasion of NSCLC cells	(153)

GBM, Glioblastoma; AML, Acute myeloid leukemia; HCC, Hepatocellular carcinoma; OC, Ovarian cancer; COAD, Colorectal adenocarcinoma; T-All, T-cell acute lymphoblastic leukemia; NSCLC, Non-small cell lung cancer.

#### 4.1.1 Inhibitors of ALKBH5

Inhibitors targeting ALKBH5 have been relatively well investigated. For example, two ALKBH5 inhibitors, 2-{[1-hydroxy-2-oxo-2-phenylethyl] sulfanyl}acetic acid and 4-{[furan-2-yl]methyl}amino-1,2-diazinane-3,6-dione, can effectively inhibit the proliferation of leukemia cells (HL-60, CCRF-CEM, and K562) at low micromolar concentrations (138). A specific ALKBH5 inhibitor ALK-04 can not only suppress the infiltration of Treg cells and MDSCs but also enhance the efficacy of anti-PD-1 therapy in inhibiting tumorigenesis (139). Imidazobenzoxazin-5-thione MV1035, a new sodium channel blocker, demonstrates significant inhibition of migration and invasion in U87 glioblastoma cell lines by reducing ALKBH5 expression (140). DDO-2728, a selective ALKBH5 inhibitor, can increase the abundance of m6A modifications in AML cells, reduce TACC3 mRNA stability, and impede cell cycle progression (141). Meanwhile, it also suppresses tumor growth in the MV4–11 xenograft mouse model (141). In addition, a novel inhibitor of ALKBH5, named TD19, has been identified to irreversibly modify residues C100 and C267, thus preventing ALKBH5 from binding to m6A-containing RNA and displays promising anti-cancer efficacy in AML and glioblastoma multiforme cell lines (142). Curcumin, a natural polyphenolic compound present in turmeric, enables to inhibition of adipogenesis by reducing the expression of ALKBH5 (143). Furthermore, an ALKBH5 inhibitor IOX1, can suppress ALKBH5 activity through a cofactor 2-oxoglutarate oxygenase competitive manner to enhance the therapeutic efficacy of anti-PD-1 treatment in preclinical glioma mice models (83). In addition, Fang et al. (144) found that a potent, selective, and cell-active inhibitor named 5-hydroxy-1-(3-(trifluoromethyl) phenyl)-1 H-Pyrazole-3-carboxylic acid (20m) can inhibit ALKBH5 in HepG2 cells and increase m6A levels.

#### 4.1.2 Inhibitors of reader proteins

An increasing number of studies on developing inhibitors of reader proteins have been conducted. The organ selenium compound Ebeselen has been reported to disrupt the interaction between the YTHDF1 m6A domain and m6A-decorated mRNA targets (145). Tegaserod, a potential inhibitor of YTHDF1, can block the direct binding of YTHDF1 with m6A-modified mRNAs and inhibit YTHDF1-regulated cyclin E2 translation, which results in reduced viability of patient-derived AML cells *in vitro (* 146). The YTHDF2 inhibitor, DC-Y13-27, can reverse the increase of ionizing radiation (IR)-induced MDSC in immunosuppressive cells and enhance the efficacy of anti-PD-L1 treatment in MC38 mouse models (147). Moreover, the specific IGF2BP1 inhibitor BTYNB is shown to downregulate the expression of Myc mRNA and suppress Melanoma and Ovarian cancer cell proliferation (148). Cucurbitacin B (CuB), a small molecule, directly targets IGF2BP1 to disrupt its recognition of m6A mRNA targets, leading to a strong correlation with cell apoptosis and immune response (149). An inhibitor of IGF2BP2 named JX5 can reduce NOTCH1 mRNA expression and trigger apoptosis to inhibit the proliferation of T-cell acute lymphoblastic leukemia (T-ALL) cells (150). In addition, Weng et al. (151) found that the IGF2BP2 inhibitor CWI1–2 suppresses Gln metabolism and impairs mitochondria function, leading to decreased ATP production in AML cells. Furthermore, a small molecule inhibitor named AE-848 can selectively disrupt the stabilization of IGF2BP3 mRNAs, thereby reducing the protein levels of c-MYC, VEGF, CDK2, CDK6, and STAT1 (152). This ultimately inhibits the progression of OC. Isoliquiritigenin (ISL), which is derived from the Chinese herb licorice, has been demonstrated to be an anticarcinogen for NSCLC (153). In terms of the mechanism, ISL can inhibit m6A modification and downregulate the expression level of IGF2BP3 in NSCLC cells, thus suppressing the proliferation, migration, and invasion of NSCLC cells (153).

In brief, the above inhibitors of both ALKBH5 and reader proteins (YTHDF1, YTHDF2, IGF2BP1, IGF2BP2, IGF2BP3) have displayed antitumor effects in different tumors, indicating their potential for the development of targeted drugs for future clinical treatment.

### 4.2 Inducible editing of ALKBH5 and reader proteins via CRISPR-Cas strategies

**Figure 4:**
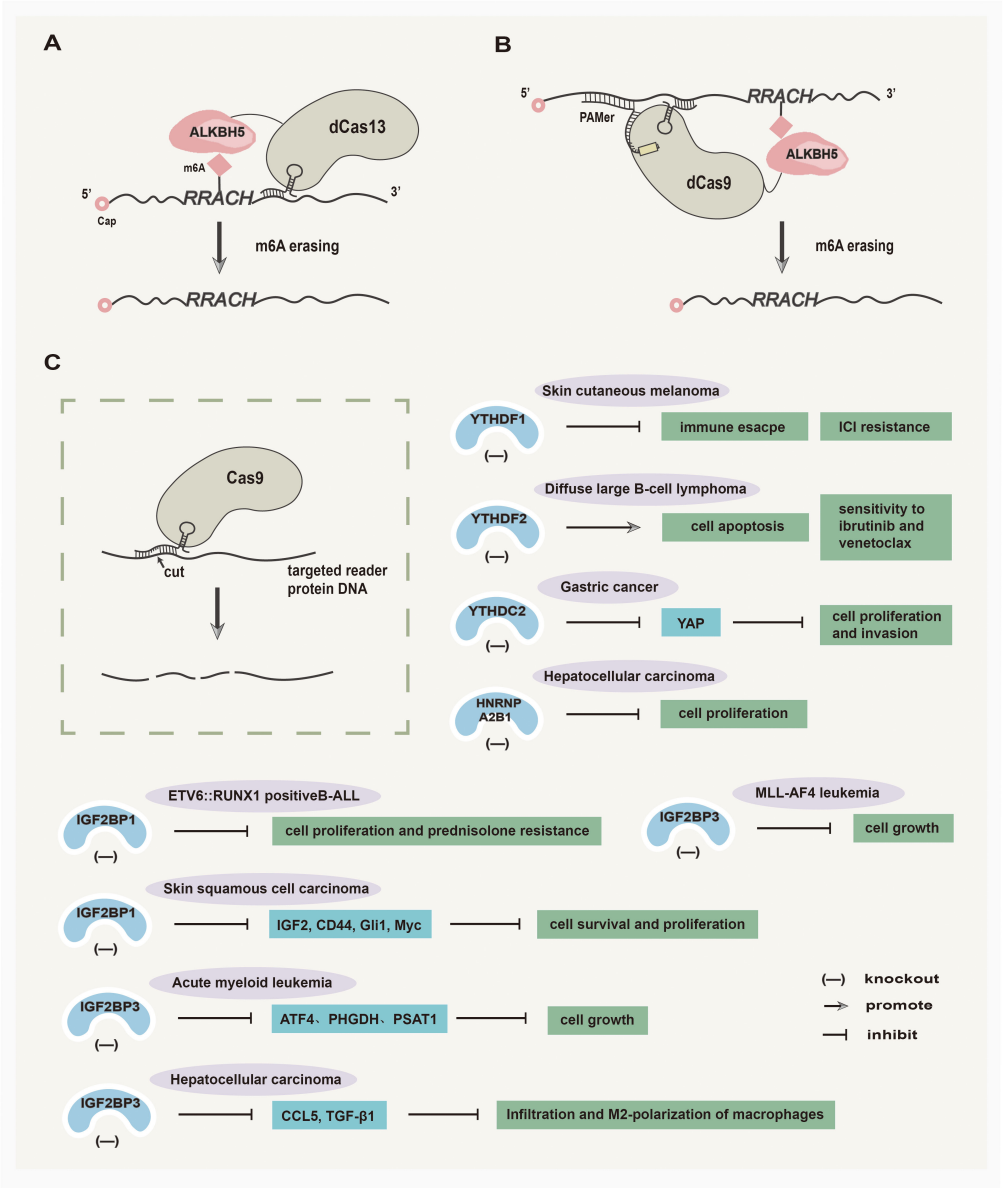
Inducible editing of ALKBH5 and reader proteins via CRISPR-Cas strategies. **(A)** Catalytically inactive Cas13 (dCas13) fused with the ALKBH5 domain removes m6A modifications. **(B)** Catalytically inactive Cas9 (dCas9) targets RNA demethylation by engineering ALKBH5. **(C)** The utilization of the CRISPR-Cas9 tool shows promises in treating various solid tumors by effectively targeting the reader proteins.

Although much drug research has targeted the proteins that regulate m6A modification, fusing these proteins to catalytically inactive CRISPR proteins has led to a novel wave of programmable RNA methylation tools (154). The inducible editing of m6A presents the potential for targeted manipulation via a site-specific manner, avoiding unwanted off-target effects (154) (**Figure 4**). Among them, CRISPR/Cas9 is a pioneering and efficient genome editing technology, which follows the specific PAM sequence (2–6 bp) on target DNA for genome editing and is widely used in the editing of ALKBH5 and reader proteins in tumors (155). In addition, another CRISPR-Cas system named CRISPR-Cas13 has been investigated for its potential in RNA-targeted editing. To be specific, the CRISPR RNA can specifically cleave RNA by interacting with the stem-loop-rich chain of uracil residues and the Cas13a protein, which offers a high-throughput and convenient technology for the development of tumor therapy (156). Here, the inducible editing of ALKBH5 and reader proteins via CRISPR-Cas strategies are summarized and discussed separately.

#### 4.2.1 Inducible editing of ALKBH5 via CRISPR-Cas strategies

In recent years, several studies have emerged regarding the inducible editing of ALKBH5 via CRISPR-Cas strategies. Liu et al. (157) have developed CRISPR/Cas9 fusions with a single chain m6A methyltransferase ALKBH5 or FTO to achieve demethylation of RNAs at site-specific, which broadens the scope of RNA engineering and facilitates mechanistic understanding of epitranscriptome in tumor research. Furthermore, Ying et al. (158) have designed a bidirectional deactivated RfxCas13d (dCasRx)-based m6A-editing platform that comprises the catalytic domains of METTL3 or ALKBH5 to manipulate the m6A demethylation of ITGA6 mRNA, thereby inhibiting BLCA cell growth.

#### 4.2.2 Inducible editing of reader proteins via CRISPR-Cas strategies

Inducible editing of reader proteins via CRISPR-Cas strategies has been extensively investigated. An exosome-mediated CRISPR/Cas9 delivery system was designed to target the oncogenic YTHDF1 gene *in vivo*, with the goal of depleting YTHDF1 to mitigate tumor progression by restoring antitumor immunity (159). Chen et al. (160) have demonstrated that CRISPR/Cas9-mediated YTHDF2 knockout can inhibit cell proliferation, induce G2/M phase cell cycle arrest, promote apoptosis, and increase the sensitivity of diffuse large B-cell lymphoma (DLBCL) to ibrutinib and venetoclax. The CRISPR/Cas9-mediated YTHDC2 knockout can effectively inhibit the translation of YAP mRNA, resulting in the suppression of gastric cancer cell viability, proliferation, and invasion (161). In addition, Sharma et al. (162) found that the CRISPR/Cas9-mediated knockout of IGF2BP1 in ETV6::RUNX1 positive B-cell Acute lymphoblastic leukemia (B-ALL) cell lines can decrease tumor cell proliferation and prednisolone resistance in Reh cell line. In skin squamous cell carcinoma (SCC), CRISPR/Cas9-mediated IGF2BP1 knockout reduces the levels of IGF2BP1-stabilized mRNAs, including IGF2, CD44, Gli1, and Myc, subsequently leading to the inhibition of skin SCC cell survival and proliferation (163). Furthermore, the CRISPR/Cas9-mediated IGF2BP3 knockout makes AML cells more sensitive to serine and glycine (SG) deprivation, and ultimately demonstrates that IGF2BP3 silencing combined with dietary SG restriction effectively inhibits AML (164). In MLL-AF4 leukemia, the deletion of IGF2BP3 mediated by CRISPR/Cas9 sensitizes MLL-AF4 leukemia to the effects of menin-MLL inhibition on cell growth and leukemic initiating cells *in vitro*, which shows potent antileukemic effects (165). In HCC, CRISPR/Cas9 technology was used to delete IGF2BP3 in HCC cells, leading to the suppression of macrophage infiltration and M2-polarization and thus inhibiting the growth of HCC (166). Hao et al. (167) have demonstrated that CRISPR/Cas9-mediated HNRNPA2B1 deletion in HCC cells can slow the progression of HCC.

In conclusion, the application of CRISPR-Cas systems to edit ALKBH5 and reader proteins shows the potential for anticancer drug development. Further exploration will lead to a deeper understanding of the mechanism for inducible editing of ALKBH5 and reader proteins via CRISPR-Cas strategies and improve editing efficiency.

### 4.3 Nanoparticle platforms

With the development of innovative nanotechnology, nanoparticles (NPs), ranging in size from 1 to 100 nm, have been fabricated for extensive applications in fields such as biomedical diagnostics, drug delivery, and anticancer therapy (168, 169). Within the field of epigenetics, NP platforms that target ALKBH5 and reader proteins have emerged as a potentially promising approach in anticancer drug development (170).

#### 4.3.1 Nanoparticle platforms targeting ALKBH5

Recently, research on NP platforms targeting ALKBH5 has mainly focused on CRC. Zhai et al. (171) discovered that vesicle-like nanoparticle-encapsulated ALKBH5-small interfering RNA can potentiate an-PD1 therapy efficacy in inhibiting CRC growth by promoting antitumor immunity. Furthermore, Wu et al. (172) have designed and synthesized the ALKBH5 mRNA-loaded folic acid-modified exosome-liposome hybrid nanoparticles to suppress CRC progression in preclinical tumor models.

#### 4.3.2 Nanoparticle platforms targeting reader proteins

At present, the development of NP platforms targeting reader proteins is being intensively explored. Chen et al. (173) have developed a photosensitive and dual-targeting nanoparticle system (M.RGD@Cr-CTS-siYTHDF1NPs) capable of delivering small interfering RNA YTHDF1 for silencing YTHDF1, leading to a pro-immunogenic tumor microenvironment (TME) state and the inhibition of immunosuppressive tumor immune microenvironment (TIME). A vesicle-like nanoparticles (VNPs)-encapsulated YTHDF1-siRNA (VNPs-siYTHDF1) was utilized to target YTHDF1, enhancing anti-PD1 efficacy in MC38 (MSI-H-CRC) and overcoming anti-PD1 resistance in CT26 (MSS-CRC) (174). In non-alcoholic steatohepatitis-associated HCC (NASH-HCC), YTHDF1 was targeted by lipid nanoparticles (LNPs)-encapsulated small-interfering YTHDF1, which can promote antitumor immunity by suppressing EZH2-IL-6 axis (175). Furthermore, Zhang et al. (176) found that LNPs targeting YTHDF1 significantly inhibit HCC stemness and enhance the efficacy of tyrosine kinase inhibitors lenvatinib and sorafenib in HCC *in vivo*. In addition, Chen et al. (177) have discovered that using LNPs encapsulating siRNA and indocyanine green (ICG) to target YTHDF3 can suppress the progression of BC and its metastasis to the lungs.

Based on the above studies, targeting ALKBH5 and reader proteins with NPs may serve as an “epigenetic drug” in the development of oncology agents, thus providing a new strategy for antitumor therapy. In the future, it is worth exploring the impact of NP platforms targeting IGF2BP family proteins on tumor growth and the underlying mechanisms, which may offer new insights into antitumor treatment.

### 4.4 CAR technology

Utilizing chimeric antigen receptor (CAR) technology in adoptive cell therapy represents a highly advanced engineering platform for tumor immunotherapy, especially leukemia and lymphoma (178). Specifically, CAR immune cells are typically derived from the peripheral blood of patients, genetically modified to express CARs *in vitro*, and then expanded before being reinjected into patients (178). Extensive research has been conducted on CAR-T, CAR-NK, and CAR-macrophage therapies, with CAR-T therapy being the most widely studied (179). The TIME consists of innate immune cells, adaptive immune cells, extracellular immune factors, and cell surface molecules, which play a crucial role in tumorigenesis and development (180). Increasing evidence has shown that ALKBH5 and reader proteins can regulate the immunosuppressive TIME in different tumors (60, 181). For example, ALKBH5-mediated changes in m6A modification may regulate the expression of Mct4/Slc16a3 and lactate content in the CRC microenvironment, as well as the composition of tumor-infiltrating Treg and myeloid-derived suppressor cells (139). Ying et al. (182) reported that in BC, the high level of YTHDF1 positively correlates with the infiltration of memory CD4+T cells activation and negatively correlates with the infiltration level of NK cells activated monocytes and macrophages M1. Furthermore, YTHDF1 silence can increase MHCII expression and interleukin-12 (IL-12) secretion, promoting the infiltration of CD4^+^ and CD8^+^ T cells and mediating overexpression of IFN-γ receptor 1 as well as JAK/STAT1 signaling pathway, thus recovering sensitivity to anti-tumor immunity in GC (183). Moreover, Bing et al. (184) found that the overexpression of IGF2BP1 can suppress the CD8+T cell-mediated antitumor response and augment the stability of PD-L1 mRNA to promote immune escape in GC cells. The above studies suggest that targeting ALKBH5 and reader proteins may relieve the TIME immunosuppression and enhance the efficacy of CAR immune cell therapy. Hence, the modulation of m6A modification in CAR immune cells may represent a potentially promising strategy to enhance anti-tumor immune responses.

## 5 Conclusion and perspective

Over the years, the underlying interaction mechanisms between demethylase ALKBH5 and reader proteins in tumorigenesis and development have been explored. However, the involved mechanisms are diverse and intricate. In this review, ALKBH5 and reader proteins, including their structures, functions, and synergistic mechanisms are retrospected. The interactions between ALKBH5 and reader proteins could modulate stages of targeted RNA processing in tumors, leading to alterations in cellular behaviors. Interestingly, in NSCLC (34, 35, 84), CRC (27, 89, 92), PC (70, 94), and Osteosarcoma (26, 77, 100, 101), although ALKBH5 can act as both a promotor and suppresser of tumorigenesis and development, it is becoming increasingly apparent that the effects of ALKBH5 on cancer cells may depend on different target genes regulated by ALKBH5. The controversial role of ALKBH5 in the same/different cancers may be attributed to the existence of genetic and epigenetic heterogeneities among the cancer cell lines and primary tumor specimens utilized by different research groups. Besides, the cooperative mechanisms of ALKBH5 and m6A reader proteins in promoting the expression of associated targets and the consequent alterations in the biological characteristics of tumors are investigated. Based on the m6A modification mechanisms, variations in the expression levels or types of reader proteins in different tumor tissues may have an impact on ALKBH5’s regulation of its downstream targets and its role in cancer. In osteosarcoma tissue, YTHDF1 and YTHDF2 selectively bind to different m6A-modified targets in the ALKBH5 regulatory network, ultimately exerting a synergy to upregulate YAP expression that suppresses tumor growth (101). While in NSCLC, YTHDF1 and YTHDF2 competitively bind to the same m6A-modified target in the ALKBH5 regulatory network, resulting in an inverse influence on YAP expression (84). The higher expression level of YTHDF1 binds to methylated YAP mRNA to promote its translation, and ALKBH5-mediated YAP demethylation can upregulate YAP expression to inhibit tumor growth (84), showing that a deeper comprehension of the interaction mechanisms between ALKBH5 and reader proteins could provide more precise and ideal targets for the development of tumor inhibitors. Furthermore, up to now, much of the evidence shows that the dysregulated ALKBH5 modulates its downstream target expression via an m6A-YTHDFs/IGF2BPs-dependent manner, while the mechanisms underlying the interaction between ALKBH5 and other reader proteins, including YTHDCs, HNRNPs, and newly identified reader proteins, remain inadequately elucidated and deserve further investigation.

In addition, the development of ALKBH5 and reader protein inhibitors is gradually becoming a promising anti-tumor strategy. However, these inhibitors still face numerous challenges, and there is a relative shortage of inhibitors that have entered clinical trials, highlighting the need to carry out preclinical and clinical trials to investigate safety profiles, therapeutic effectiveness, and pharmacokinetics. Additionally, there is a need for a thorough investigation into the potential of combining the inhibitors of ALKBH5 and reader protein with existing antineoplastic agents to enhance the efficiency of antitumor therapy. Besides, the advancement of both the m6A editing system and encapsulation of m6A modification molecules into NP platforms holds great prospects for tumor therapy. Recent research has discovered that NPs are associated with the dysregulation of certain cellular processes and diseases when they enter the body through the digestive system, respiratory system, and skin tissue (185). Up to now, there has been a growing number of reports on NPs targeting ALKBH5 and YTHDF family proteins to influence tumor growth. Nevertheless, relatively few reports have focused on the effects of NP platforms targeting the IGF2BP family proteins on tumor development and the underlying specific mechanisms, which need further exploration. Additionally, the potential development of the therapy strategy that combines targeting ALKBH5 and reader proteins with CAR technology may represent a breakthrough in cancer immunotherapy in the future. Meanwhile, it is worthwhile to investigate whether targeting ALKBH5 and reader proteins in immune cells derived from patients can enhance CAR expression, potentially leading to improved efficacy of CAR immune cell therapy.

With increasing research on m6A modification in tumors, ALKBH5 and reader proteins can be used as novel biomarkers for tumor diagnosis and prognosis, even providing new opportunities for developing anti-tumor drugs. The deficiencies mentioned above can be progressively improved in future studies.

## Glossary

m6A: N6-methyladenosine
mRNA: Messenger RNA
miRNA: MicroRNA
lncRNA: Long non-coding RNA
circRNA: Circular RNA
tRNA: Transfer RNA
carRNA: Chromosome-associated regulatory RNA
3’UTR: 3’untranslated region
MTC: Methyltransferase complex
ALKBH5: Alpha-ketoglutarate-dependent dioxygenase alkB homolog 5
FTO: Fat mass and obesity-related protein
HNRNPs: Heterogeneous nuclear ribonucleoprotein
IGF2BPs: Insulin-like growth factor 2 mRNA binding protein
YTHDFs: YT521-B homology domain-containing family proteins
YTHDCs: YT521-B homology domain containing
eIF3: Eukaryotic starting factor 3
Treg: Regulatory T cell
MDSCs: Myeloid-derived suppressor cells
PD-1: Programmed cell death receptor 1
PD-L1: Programmed cell death 1 ligand 1
TME: Tumor microenvironment
TIME: Tumor immune microenvironment
2OG: 2-oxoglutarate
DBSH: Double-stranded β-helix
HPSCC: Hypopharyngeal squamous cell carcinoma
NSCLC: Non-small cell lung cancer
HNSCC: Head and neck squamous cell carcinoma
KL: KRAS mutations and LKB1 tumor-suppressor gene
CTCF: CCCTC-binding factor
EMT: Epithelial-mesenchymal transition
BUB1B: BUB1 mitotic checkpoint serine/threonine kinase B
CSCs: Cancer stem cells
CRC: Colorectal cancer
HCC: Hepatocellular carcinoma
NASH-HCC: Non-alcoholic steatohepatitis- associated HCC
GC: Gastric cancer
YY1: Yin-Yang 1
IL: Interleukin
IFN-γ: Interferon-γ
PC: Pancreatic cancer
PanNETs: Pancreatic neuroendocrine tumors
CC: Cervical cancer
CESC: Cervical squamous cell carcinomas
HPV: Human papillomavirus
BLCA: Bladder cancer
ICC: Intrahepatic cholangiocarcinoma
EOC: Epithelial ovarian cancer
LN: Lymph node
FAK: Focal adhesion kinase
OS: Osteosarcoma
TMA: Tissue microarray
MM: Multiple myeloma
BC: Breast cancer
AML: Acute myeloid leukemia
CNS: Central nervous system
GBM: Glioblastoma multiforme
TAM: Tumor-associated macrophage
CAR: Chimeric antigen receptor
NPs: Nanoparticles
CRISPR: Clustered regularly interspaced short palindromic repeats
T-ALL: T-cell acute lymphoblastic leukemia
dCas13: Catalytically inactive Cas13
dCas9: Catalytically inactive Cas9
DLBCL: Diffuse large B-cell lymphoma
OC: Ovarian cancer
SG: Serine and glycine.
